# *Yersinia enterocolitica* and *Yersinia pseudotuberculosis* Detection in Foods

**DOI:** 10.4061/2011/735308

**Published:** 2011-10-05

**Authors:** H. Fukushima, S. Shimizu, Y. Inatsu

**Affiliations:** ^1^Shimane Prefectural Institute of Public Health and Environment Science, Izumo 690-0122, Japan; ^2^Food Hygiene Laboratory, National Food Research Institute, Tsukuba 305-8642, Japan; ^3^Food Safety Laboratory, Faculty of Fisheries Sciences, Hokkaido University, Hakodate 041-8611, Japan

## Abstract

*Yersinia enterocolitica* and *Y. pseudotuberculosis* which can cause yersiniosis in humans and animals are thought to be significant food-borne pathogens and be important as hygiene indicator in food safety. The pathogenic *Y. enterocolitica* serotypes/biotypes are O:3/4 and 3 variant VP negative, O:5, 27/2, O:8/1b, and O:9/2, have been reported worldwide. *Y. pseudotuberculosis* is distributed less widely than *Y. enterocolitica*. Isolation methods usually involve selective and recovery enrichment of the food sample followed by plating onto selective media, confirmation of typical colonies and testing for virulence properties of isolated strains. Recently, DNA-based methods, such as PCR assays, have been developed to detect pathogenic *Y. enterocolitica* and *Y. pseudotuberculosis* in foods more rapidly, and sensitivity than can be achieved by conventional culture methods. This paper reviews commercially available conventional and PCR-based procedures for the detection of pathogenic *Yersinia* in food. These methods are effective as the isolation and detection methods to target pathogenic *Y. enterocolitica* and *Y. pseudotuberculosis* in foods.

## 1. Overview

Food-borne pathogenic *Yersinia* (*Y. enterocolitica *and *Y. pseudotuberculosis*) is facultative anaerobic, gram-negative *Enterobacteriaceae* and is isolated frequently from soil, water, animals, and foods [1–4]. *Y. enterocolitica* causes human infections whose symptoms include diarrhea, terminal ileitis, mesenteric lymphadenitis, arthritis, and septicemia. *Y. pseudotuberculosis* causes mesenteric lymphadenitis, diarrhea, and septicemia in humans. As a psychrophilic organism, *Yersinia* is able to grow at 4°C, and cold chain food products could offer a potential food safety hazard [3, 5, 6]. The pathogenic *Y. enterocolitica* serotypes/biotypes are O:3/4 and 3 variant VP negative, O:5, 27/2, O:8/1b, and O:9/2 have been reported worldwide [7, 8]. In Japan, O:3/3 variant VP negative is the most frequent cause of human yersiniosis [8]. In the United States, despite declining incidences of serotype O:8/1b infections, O:3/4 and O:5, 27/2 infections are on the increase [7]. In Europe, Serotype O:3 and O:9 infections account for over 90% of *Y. enterocolitica *infections. *Y. pseudotuberculosis* is distributed less widely than *Y. enterocolitica* and, although frequently isolated from animals, is rarely isolated from soil, water, and food [9–12]. 

A large outbreak of *Y. pseudotuberculosis *infection has been reported in Japan [13, 14]. In the Far East including Japan, *Y. pseudotuberculosis* various serotypes (1b, 2a, 2b, 2c, 3, 4a, 4b, 5a, 5b, and all that) are isolated from patients with exanthematous systemic infection such as fever, and almost strains isolated produce a superantigenic toxin-designed YPMa encoded by *ypmA* gene [14–16]. In Europe, serotypes (1a, 1b, and 3) have been isolated from patients with gastroenteric symptoms and have an extremely low frequency of isolation [16].

It is therefore important to isolate and identify and differentiate food-borne pathogenic *Yersinia *from nonpathogenic *Yersinia* strains. Isolation methods usually involve enrichment of the food sample followed by plating onto selective media, confirmation of typical colonies, and testing for virulence properties of isolated strains [17]. This method is an effective method which may be employed to *Yersinia enterocolitica *and *Y. pseudotuberculosis *in foods. The procedure has been used especially to detect the pathogenic *Y. enterocolitica *and *Y. pseudotuberculosis* in Japan. 

## 2. Procedures Currently to Quantify and Confirm *Yersinia* sp. in Food

**Figure 1:**
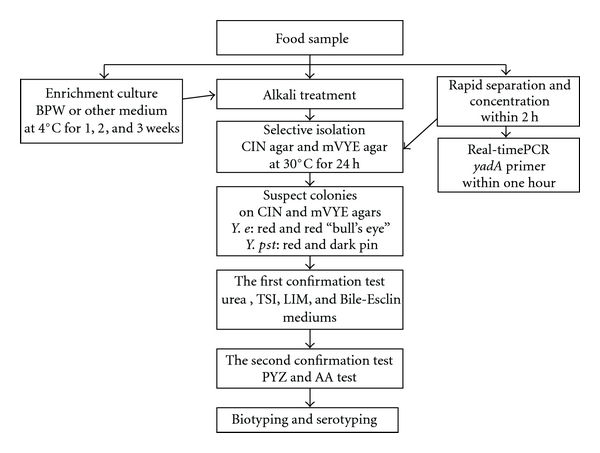
Optimal protocol for detection and identification of *Y. enterocolitica *and *Y. pseudotuberculosis* from foods.

The presence of *Y. enterocolitica *and *Y. pseudotuberculosis* in food can be determined quantitatively by a direct culture on selective agar plates. However, confirmatory tests require a combination of cold enrichment, selective enrichment, and subculture on selective agar plates. A conventional protocol for detection and identification of *Y. enterocolitica *and *Y. pseudotuberculosis* from foods is shown in Figure 1. Suspect food samples must however be pretreated to enable successful analysis.

### 2.1. Pretreatment of Foods

Pretreatment starts with the homogenizing the food sample (25 g) in a stomacher for 2 min with 225 mL of phosphate-buffered saline (PBS) or other cold enrichment medium (see below). The resulting homogenate is used for the direct culture, enrichment culture experiments. For rapid separation and concentration, a 25 g food sample is mixed with 225 mL of 0.02% Tween 20-buffered peptone water (BPW) in a plastic bag (Stomafilter P type; Gunze, Tokyo, Japan), containing a Teflon cloth (40 mesh) on the inside and homogenized in a stomacher for 2 min [18].

### 2.2. Enumeration of *Yersinia* sp. by Direct Culture Method

For this procedure, an aliquot of homogenate is inoculated onto selective agar plates (see below) after treatment with an alkali [9, 19]. Alkaline treatment can be achieved by mixing 0.5 mL of homogenate with 0.5 mL of 0.72% KOH in 0.54% NaCl for 30 sec. *Yersinia* is able to resist weak alkaline treatment, and this property is used to select the organism while suppressing background flora such as *Pseudomonas, Proteus* and *Serratia* [20]. It is reported that* Y. enterocolitica *serotypes O:3, O:5, 27, O:8, and O:9 and *Y. pseudotuberculosis *serotype 5a strains in the artificially contaminated pork samples showed comparatively high resistance to KOH, and all *Yersinia *strains were recovered from the pork samples contaminated with more than 10^2^ cells per g after direct KOH treatment, without enrichment [9]. However, food samples with low contamination (less than 10^2^ cells per g) require an enrichment procedure for successful recovery of *Yersinia*.

### 2.3. Cold and Selective Enrichment for Recovering *Yersinia* sp. from Food Samples with Low Contamination Levels

For cold enrichment, an inoculated medium (examples shown below) is incubated at 4°C for three weeks. After 1, 2, and 3 weeks, 0.5 mL of the medium is treated with KOH and inoculated onto selective agar plates. This procedure is useful for enrichment of *Y. enterocolitica *and* Y. pseudotuberculosis*. Being psychrophilic, *Yersinia* can grow at 4°C. However if the medium has low selectivity, environmental *Yersinia *species and other bacteria may also multiply during enrichment [17]. Alkali treatment of the medium helps reduce such non*Yersinia* background flora. Cold enrichment media used for detection of *Yersinia* in food and water samples are

1. phosphate-buffered saline (PBS; 880 mL of 0.061 M Na_2_HPO_4_, 120 mL of 0.061 M KH_2_PO_4_, and 0.85% NaCl) [21],
2. PBS with 1% mannitol and 0.15% bile salts (PMB) [22],
3. PBS with 0.5% peptone and 1% sorbitol, 0.15% bile salts (PSB) [23],
4. PBS with 0.25% peptone and 0.25% mannitol (PMP) [5],
5. buffered peptone water (BPW, Merck, Germany).

An alternative to cold enrichment is selective enrichment. Selective enrichment uses media containing antimicrobial agents. Several selective enrichment media for isolation of *Y. enterocolitica *at higher temperatures have been developed [17]. Generally, cold enrichment yields higher recovery rates of pathogenic *Y. enterocolitica *than selective enrichment. Moreover, an effective selective enrichment system for *Y. pseudotuberculosis* has not been developed, so its current selective enrichment procedures are especially low. 

Nevertheless, in case of outbreaks, selective enrichment procedures for isolation of pathogenic *Y. enterocolitica* are useful for rapid detection and confirmation of the pathogen. In such cases, 9 mL of Irgasan-ticarcillin-potassium chlorate (ITC, Merck, Darmstadt, Germany) is inoculated with 1 mL of medium from cold enrichment and aerobically incubated at 25–30°C for 48 hr [22].

### 2.4. Rapid Separation and Concentration of *Yersinia* from Food Samples for Cell Counting and PCR [18]

**Figure 2:**
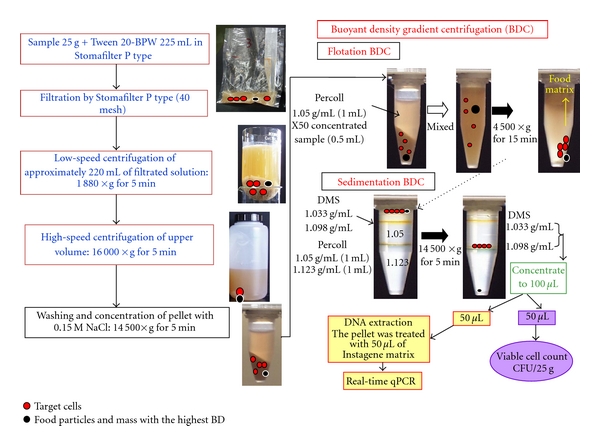
Optimal protocol for rapid separation and concentration of food-borne pathogens in food samples using filtration, centrifugation, and BDC prior to quantification by viable-cell counting and real-time PCR.

A conventional protocol for rapid separation and concentration of food-borne pathogens in food samples using filtration, centrifugation, and buoyant density centrifugation (BDC) prior to quantification by viable-cell counting and real-time PCR is shown in Figure 2. A 25 g food sample is mixed with 225 mL of 0.02% Tween 20-BPW in a small plastic bag (Stomafilter P type) and homogenized in a stomacher for 2 min. Approximately 220 mL portions of filtered solutions of the homogenates are placed in sterilized 350 mL glass tubes and centrifuged at 1,880 ×g for 5 min at room temperature, using a swing rotor. The upper portion is transferred to a sterilized 500 mL plastic tube and then centrifuged at 16,000 ×g for 5 min at room temperature. The pellet is then suspended in 1.5 mL of 0.15 M NaCl and centrifuged at 14,500 ×g with a bench-top centrifuge for 5 min at room temperature. The resultant pellet is harvested and used for the second step.

The second step is flotation and sedimentation BDC for purification of food-borne pathogens. In the flotation assay, 0.5 mL portions of sample suspensions are mixed with 1 mL of a 1.050 g/mL Percoll solution (Pharmacia Biotech, Sweden) and centrifuged at 4,500 ×g for 15 min at room temperature. The upper portion, including the food matrix, is carefully removed. For the sedimentation assay, the bottom portion (about 0.5 mL), including organisms, food particles, and the mass with the highest buoyant density, is homogenized and then placed on top of two layers (0.6 mL of a 1.050 g/mL Percoll solution and 0.6 mL of a 1.123 g/mL Percoll solution) in a 1.5 mL microtube to which two density markers (orange for 1.033 g/mL and green for 1.098 g/mL) are added. The preparations are centrifuged at 14,500 ×g for 5 min at room temperature, and then using sterile 1-mL pipettes, about 1 mL is taken from the interface between the two density makers and divided into two samples. The sample is added to 1 mL of 0.15 M NaCl in a 1.5 mL microtube.

The preparations are then centrifuged at 14,500 ×g for 5 min. The bottom portions (0.5 mL) are resuspended with 1 mL of 0.15 M NaCl and then centrifuged at 14,500 ×g for 5 min. Each pellet is used for viable-cell counting and DNA extraction with InstaGene matrix (Bio-Rad). One portion of the sample is resolved with 50 *μ*L of 0.15 M NaCl, and then viable-cell counts (CFU/g), which are obtained by culturing each dilution (10 *μ*L) using selective agar plates, are determined for these BDC-lysate pellets (50 *μ*L). The other portion is treated with 50 *μ*L of InstaGene matrix for DNA extraction prior to real-time qPCR by using *yadA* primer for pathogenic *Y. enterocolitica* and *Y. pseudotuberculosis*. The total volume of 25 g food sample is reduced to 0.1 mL, and the target organisms in the sample are theoretically concentrated 250-fold within 2 hr.

### 2.5. Isolation of *Yersinia* Using Selective Agar Media

Isolation of *Yersinia* and pathogenic* Yersinia* (pathogenic *Y. enterocolitica* and *Y. pseudotuberculosis*) can be done using Cefsulodin-Irgasan-Novobiocin agar (CIN agar, Difco, Oxoid) [20] and CIN agar containing 0.1% esculin and 0.05% ferric citrate (modified virulent *Yersinia enterocolitica *agar (mVYE agar)) [24]. 

**Figure 3:**
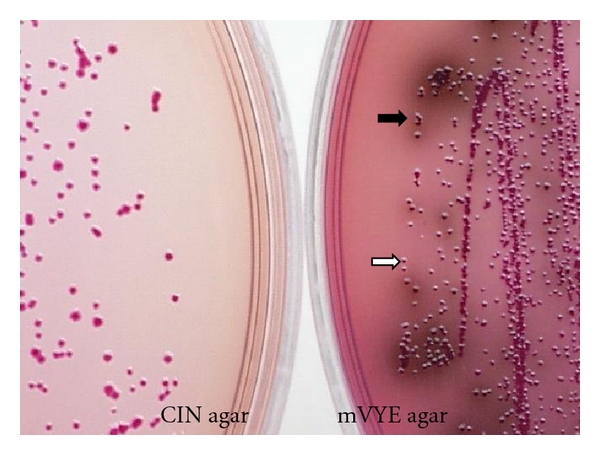
Colonies of *Y. enterocolitica* grown on CIN agar and mVYE agar incubated at 30°C for 24 h. *Yersinia* colonies on CIN form the characteristic deep red center (“bull's eye”) with a transparent margin and diameter 2–4 mm. Pathogenic *Y. enterocolitica *serotype O:3/biotype 3 variant VP^−^ (white arrow) forms red colonies and is easily differentiated from most nonpathogenic *Yersinia *organisms (black arrow) and other Gram-negative bacteria, which form dark-red colonies with a dark peripheral zone as a result of mannitol fermentation and esculin hydrolysis.

**Figure 4:**
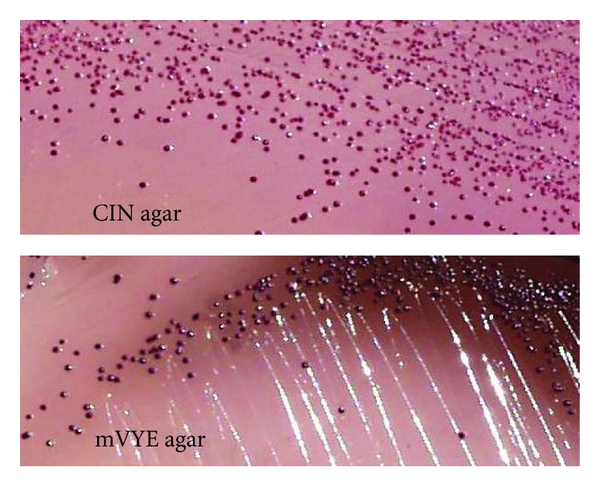
Colonies of *Y. pseudotuberculosis* grown on CIN agar and mVYE agar incubated at 30°C for 24 h. *Y. pseudotuberculosis *forms red pin colonies on CIN agar and dark-red pin colonies on mVYE agar.

CIN agar is useful to expedite the recovery of* Y. enterocolitica* and mVYE agar to differentiate virulent from avirulent isolates (Figures 3 and 4). The characteristic deep red center (“bull's eye”) with a transparent margin and diameter 2–4 mm appearance of *Yersinia* colonies on CIN incubated at 30°C for 24 hr is important for identification and is due to the presence of mannitol. *Yersinia* ferments the mannitol in the medium, producing an acid pH which gives the colonies their red color and the “bull's eye” appearance.

The greatest advantage of mVYE agar is that pathogenic *Y. enterocolitica*, which forms red colonies, is easily differentiated from most nonpathogenic *Yersinia *organisms and other gram-negative bacteria, which form dark-red colonies with a dark peripheral zone as a result of mannitol fermentation and esculin hydrolysis. *Y. pseudotuberculosis*, which forms dark pin colonies as a result of esculin hydrolysis, is easily differentiated from most nonpathogenic *Yersinia *organisms. 

The “bull's eye” colonies on CIN agar and red colonies on mVYE agar are suspected to virulent* Y. enterocolitica* (and sometimes *Y. kristensenii*). The red pin colonies on CIN agar and dark-red pin colonies on mVYE agar are suspected to *Y. pseudotuberculosis.*

### 2.6. The First Confirmation Test for Colonies from Selective Agar Media

Colonies showing typical morphology on CIN and mVYE agars (at least four colonies from each of the agar plates) are selected. The strains are confirmed according to the criteria shown in Table 1. The first confirmation test is carried out by inoculating urea broth, TSI medium, LIM medium, and Bile-esculin agar and incubating at 30°C for 24 hr.

Depending on the target organism, colonies are selected for further examination. In the case of *Y. enterocolitica, *colonies that are urea positive; lysine negative in the LIM medium; no gas formation in the TSI medium; glucose positive, sucrose positive; lactose negative (yellow slant and yellow agar column in the TSI medium) should be selected. If the targets are *Y. pseudotuberculosis*, *Y. enterocolitica* biotype 3 VP^−^, and sucrose negative/serotype O:3, colonies that are glucose positive, sucrose negative, and lactose negative (yellow slant and red agar column in the TSI medium) should be selected for further examination. It should be noted that pathogenic *Y. enterocolitica* strains (serotypes O:3, O:5, 27, O:8, and O:9) are esculin negative while *Y. pseudotuberculosis* strains and nonpathogenic *Y. enterocolitica* strains (biotype 1A/other numerous serotypes) are esculin positive.

### 2.7. The Second Confirmation Test from the First Confirmation Test

Strains suspected as pathogenic *Y. enterocolitica* and *Y. pseudotuberculosis* by the first confirmation tests are selected for the second confirmation tests (pyrazinamidase test [25] and autoagglutination test [26]). For pyrazinamidase test, the strain is inoculated onto pyrazinamidase test agar slants (see below) and incubated at 30°C for 48 hr. For autoagglutination test, the strain is incubated in Trypticase soy broth (TSB; BBL) (or MR-VP medium; Difco) at 25°C and 37°C for 24 hr.

Pyrazinamidase test agar.
Trypticase soy agar (Difco) (30.0 g).
Yeast extract (Difco) (3.0 g).
Pyrazinecarboxamide (Merck) (1.0 g).
Tris-maleate (0.2 M, pH6) to (1,000 mL).
The 5 mL portions of culture medium are autoclaved and cooled to make slants.

**Figure 5:**
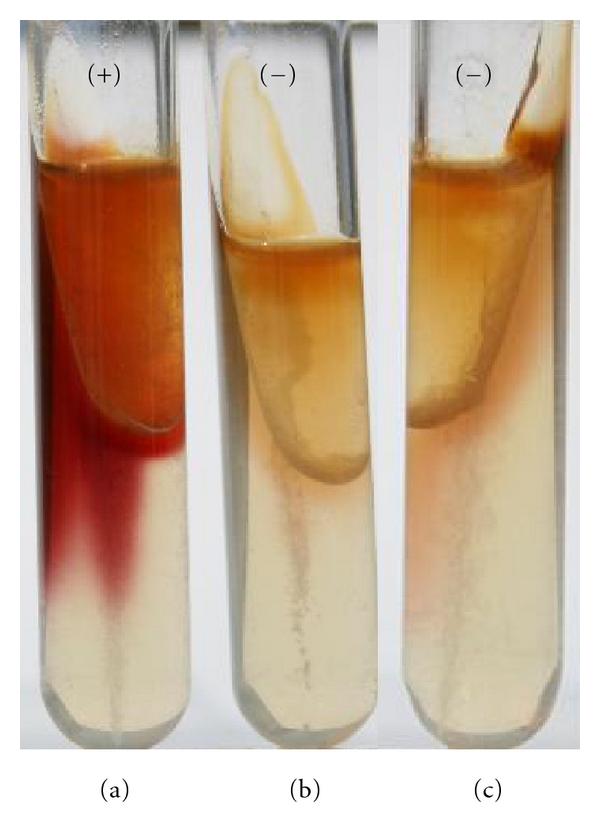
Pyrazinamidase test. A brownish pink color indicates formation of pyrazinic acid and is a positive pyrazinamidase reaction. *Y. enterocolitica* serotype O:5/biotype 1A (a) is positive and *Y. enterocolitica *serotype O:3/biotype 3 variant VP^−^ (b) and *Y. pseudotuberculosis* serotype 4b (c) are negative reaction.

**Figure 6:**
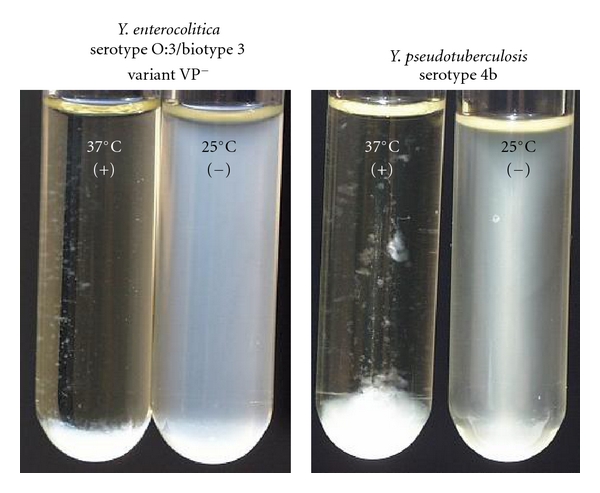
Autoagglutination test. Virulent plasmid-positive strains of *Y. enterocolitica* and *Y. pseudotuberculosis *produced outer membrane protein and autoagglutinate when were incubated in TSB or MR-VP medium at 37°C.

The pyrazinamidase test is to date the chromosomal phenotypical criterion to distinguish potentially pathogenic from nonpathogenic strains. Pathogenic *Y. enterocolitica* and *Y. pseudotuberculosis* show negative reactions, and nonpathogenic *Yersinia *strains show positive reactions which turn brownish pink in the presence of ferrous salts (Figure 5). Autoagglutination test is positive on the plasmid for *Yersinia* virulence- (pYV-) positive strains of *Y. enterocolitica* and *Y. pseudotuberculosis *which are incubated at 37°C but not at 25°C (Figure 6). The pYV lost strains which are subcultured, especially at 37°C, and stored, show negative reactions.

### 2.8. Further Biochemical and Serological Confirmation

Pure strains of suspected pathogenic *Y. enterocolitica* and *Y. pseudotuberculosis* are prepared on blood agar or other nutrient agar. The strains are investigated oxidase activity (negative), carried out Gram staining (negative). Then the strains are performed biotyping of *Y. enterocolitica* or genetic grouping of *Y. pseudotuberculosis* according to the criteria shown in Table 1. The serotyping is carried out by slide agglutination using commercial antisera O:3, O:5, O:8, and O:9 for *Y. enterocolitica* (Denka Seiken, Tokyo, Japan), and antisera O:1, O:2, O:3, O:4, O:5, and O:6 (Denka Seiken) for *Y. pseudotuberculosis.* The serotype and subserotype of *Y. pseudotuberculosis* are carried out possibly by O-genotyping using O-antigen gene cluster-specific PCRs [27].

The following parameters can be used to distinguish between *Y. enterocolitica *and other *Yersinia* species: sucrose (positive), rhamnose (negative), melibiose (negative), ornithine decarboxylase (positive) and Voges-Proskauer (VP) positive. However, VP and/or sucrose-negative strains of *Y. enterocolitica* [18, 28, 29] and melibiose-negative strains of *Y. pseudotuberculosis* [16] may occur. 

*Y. enterocolitica* serotype O:3/biotype 4 is distributed all over the world and is the dominant human pathogenic strain in western countries. However, serotype O:3/biotype 3 variant VP^−^ is the dominant human pathogenic strain in China, Taiwan, and Japan, and serotype O:3/biotype 3VP^−^, sucrose negative (S^−^) is also reported in Japan. Serotype O:5,27, which is reported in the USA, China, and Japan, and Serotype O:9, from the Nordic countries, China, and Japan, belong to biotype 2. Serotype O:8 from the USA and Japan belongs to biotype 1B. Biotype 1A comprises numerous serotypes which have not been associated with human illness and are common in food and the environment. 

*Y. pseudotuberculosis* strains belong to genetic groups 1 to 6 and serotypes 1a, 1b, 1c, 2a, 2b, 2c, 3, 4a, 4b, 5a, 5b, 6, 7, 8, 9, 10, 11, 12, 13, 14, and 15. The genetic group 3 (Far Eastern systemic-pathogenicity type)/serotypes 1b, 1c, 2a, 2b, 2c, 3, 4a, 4b, 5a, 5b, 6, 7, 8, 10, and 15 are the human pathogens in Japan, China, and Korea. The genetic group 2 (European gastroenteric-pathogenicity type)/serotypes 1a and 1b and genetic group 5/serotype O:3 are the human pathogens in western countries. Although most strains of *Y. pseudotuberculosis* are melibiose positive, genetic group 4/serotypes O:1, O:5, O:6, O:7, O:9, O:10, O:11, and O:12 (nonpathogenic strains), which are distributed in the environment of Japan, and genetic group 5/serotype O:3 are melibiose negative [16].

### 2.9. Molecular Detection by PCR for Rapid Detection of *Y. enterocolitica*

Using PCR, pathogenic* Y. enterocolitica* can be detected in samples rapidly and with high specificity and sensitivity [17]. Several PCR assays have been developed to detect pYV-positive *Y. enterocolitica* and *Y. pseudotuberculosis* in clinical, food, and environmental samples. Many of these samples use primers targeting the *yadA *or* virF *gene located on pYV. Because of possible plasmid loss on subculture and storage [30], PCR methods targeting chromosomal virulence genes have also been created for environmental samples. The *ail *gene, located in the chromosome of pathogenic *Y. enterocolitica *strains, and *inv* gene, located in the chromosome of *Y. pseudotuberculosis* strains, are the most frequently used targets. Multiplex PCR method using a mixture of primers against *inv* (5′-TAAGGGTACTATCGCGGCGGA-3′ and 5′-CGTGAAATTAACCGTCACACT-3′)*, ail *(5′-ACTCGATGATAACTGGGG AG-3′ and 5′-CCCCCAGTAATCCATAAAGG-3′), and *virF *(5′-TCATGGCAG AACAGCAGTCAG-3′ and 5′-ACTCAT CTTACCATTAAGAAG-3′) [31] has been designed to detect *Y. enterocolitica *and *Y. pseudotuberculosis *in food and water [32].

Real-time PCR is a powerful advancement of the basic PCR technique. At present, the most popular real-time PCR assays are based on “Taqman” and “SYBR Green” approaches. The Taqman system is a 5′-nuclease assay that utilizes specific hybridization of a dual-labelled Taqman probe to the PCR product. The SYBR Green system is based on the binding of the fluorescent SYBR Green dye to the PCR product [33]. Chromosomally encoded *ail* [34] and *yst* [35] genes, the plasmid-borne *yadA* gene [36, 37] and a *Yersinia*-specific region of the 16S rRNA gene [37, 38] have been used in real-time PCR. 

**Figure 7:**
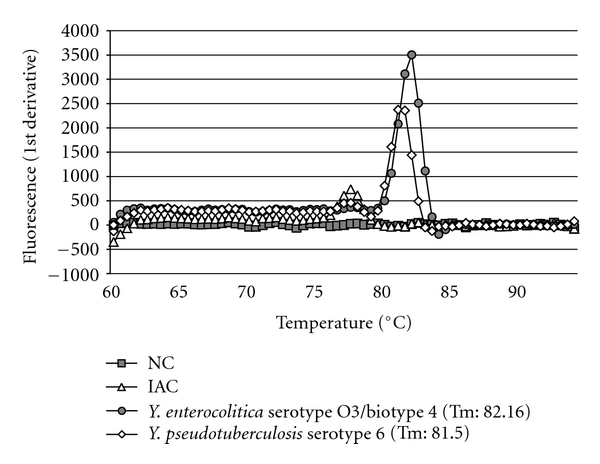
*Y. enterocolitica *and *Y. pseudotuberculosis *dissociation curve in SYBR Green real-time PCR assay.

Pathogenic *Y. enterocolitica *and *Y. pseudotuberculosis *strains yield positive PCR products from the *yadA *gene [39]. Using SYBRGreen real-time PCR assay, the *Tm *values of this *yadA *primer pair (yadA-F1757: 5′-ACGAGTTGACAAAGGTTTAGCC-3′ and yadA-R1885: 5′-GAACCAACCGCTAATGCCTGA-3′) are also different between the pathogenic *Y. enterocolitica *(82.2°C) and *Y. pseudotuberculosis *(81.5°C) strains (Figure 7). Therefore, this primer pair was confirmed to be useful for detection and differentiation of the two pathogenic *Yersinia *species.

## 3. Conclusion

*Yersinia enterocolitica* and *Y. pseudotuberculosis* continue to be important in food safety. While *Yersinia* can survive in many types of food, there is no much information about its the prevalence. This paper covers commercially available conventional and PCR-based procedures for the detection of pathogenic *Yersinia* in food. These methods are effective as the detection methods to target for pathogenic *Y. enterocolitica *and *Y. pseudotuberculosis *in foods. However, development of rapid test methods is needed to facilitate more timely and cost-effective testing.

## Figures and Tables

**Table 1:** Confirmation characteristics of pathogenic *Y. enterocolitica* and *Y. pseudotuberculosis*.

Test	Medium	Condition	Characteristic	Reactions									
Species				*Y. enterocolitica*							*Y. pseudotuberculosis*		
The first confirmation test	Urea broth	30°C, 24 h	Urea	+								+	
	TSI		Slant/column	yellow/yellow						red/yellow		red/yellow	
			H_2_S	−						−		−	
			Gas	−						−		−	
	LIM		Lysin	−						−		−	
			Indole	d						−		−	
			Motility	+,d						+		+	
	Bile-esculin agar		Esculin	+			−			−		+	

The second confirmation test	Pyrazinamidase-test agar	30°C, 48 h	Pyrazinamidase	+			−			−		−	
	TSB	25°C & 37°C, 24 h	Autoagglutination	−			+			+	+		−

Biotype of* Y. enterocolitica *				1A	1B	2	3	3 variant VP^−^	4	3 VP^−^, S	1, 2, 3, 6	5	4
Genetic group of* Y. pseudotuberculosis *													

Biotyping test	30°C, 48 h		Lipase	+	+	−	−	−	−	−	−	−	−
			Indole	+	+	(+)	−	−	−	−	−	−	−
			Xylose	+	+	+	+	+	−	+	+	+	+
			Voges-Proskauer (VP)	+	+	+	+	−	+	−	−	−	−
			Sucrose	+	+	+	+	+	+	−	−	−	−
Differentiation test			Rhamnose	−	−	−	−	−	−	−	+	+	+
			Melibiose	−	−	−	−	−	−	−	+	−	−

Serotyping				Others	O:8	O:5, 27 O:9	O:3	O:3	O:3	O:3	O:1 to O:15	O:3	part of O:1, O:5, O:6, O:7, O:9, O:10, O:11, O:12

Pyrazinamidase test is carried out by inoculating a slant of the pyrazinamidase test agar and incubating at 30°C for 48 hours. Then 1 mL of 1% freshly prepared aqueous solution of ammonium ferric sulphate is poured on the slant. After 15 minutes, the reaction is investigated. A brownish pink colour indicates formation of pyrazinic acid and is a positive pyrazinamidase reaction.

Autoagglutination test is carried out by inoculating Trypticase soy broth (TSB; BBL) (or MR-VP medium; Difco) and incubating at 25°C and 37°C for 24 hours. The virulent plasmid-positive strain invariably autoagglutinated in TSB when grown at 37°C but did not at 25°C.
